# The association between regional transcriptome profiles and lung volumes in response to mechanical ventilation and lung injury

**DOI:** 10.1186/s12931-022-01958-2

**Published:** 2022-02-19

**Authors:** Yong Song, Seiha Yen, Melissa Preissner, Ellen Bennett, Stephen Dubsky, Andreas Fouras, Peter A. Dargaville, Graeme R. Zosky

**Affiliations:** 1 Menzies Institute for Medical Research, College of Health and Medicine, University of Tasmania, Hobart, TAS Australia; 2 Department of Mechanical and Aerospace Engineering, Monash University, Melbourne, VIC Australia; 3 4Dx Limited, Melbourne, VIC Australia; 4 Tasmanian School of Medicine, College of Health and Medicine, University of Tasmania, Hobart, TAS Australia

**Keywords:** Mechanical ventilation, Ventilator-induced lung injury, Sepsis, Lung inhomogeneities, RNA sequencing, Transcriptome

## Abstract

**Background:**

Lung inhomogeneity plays a pivotal role in the development of ventilator-induced lung injury (VILI), particularly in the context of pre-existing lung injury. The mechanisms that underlie this interaction are poorly understood. We aimed to elucidate the regional transcriptomic response to mechanical ventilation (MV), with or without pre-existing lung injury, and link this to the regional lung volume response to MV.

**Methods:**

Adult female BALB/c mice were randomly assigned into one of four groups: Saline, MV, lipopolysaccharide (LPS) or LPS/MV. Lung volumes (tidal volume, Vt; end-expiratory volume, EEV) were measured at baseline or after 2 h of ventilation using four-dimensional computed tomography (4DCT). Regional lung tissue samples corresponding to specific imaging regions were analysed for the transcriptome response by RNA-Seq. Bioinformatics analyses were conducted and the regional expression of dysregulated gene clusters was then correlated with the lung volume response.

**Results:**

MV in the absence of pre-existing lung injury was associated with regional variations in tidal stretch. The addition of LPS also caused regional increases in EEV. We identified 345, 141 and 184 region-specific differentially expressed genes in response to MV, LPS and LPS/MV, respectively. Amongst these candidate genes, up-regulation of genes related to immune responses were positively correlated with increased regional tidal stretch in the MV group, while dysregulation of genes associated with endothelial barrier related pathways were associated with increased regional EEV and Vt when MV was combined with LPS. Further protein–protein interaction analysis led to the identification of two protein clusters representing the PI3K/Akt and MEK/ERK signalling hubs which may explain the interaction between MV and LPS exposure.

**Conclusion:**

The biological pathways associated with lung volume inhomogeneity during MV, and MV in the presence of pre-existing inflammation, differed. MV related tidal stretch induced up-regulation of immune response genes, while LPS combined with MV disrupted PI3K/Akt and MEK/ERK signalling.

**Supplementary Information:**

The online version contains supplementary material available at 10.1186/s12931-022-01958-2.

## Introduction

Mechanical ventilation (MV) is the mainstay of treatment for patients with acute respiratory distress syndrome (ARDS). Unfortunately, MV creates excessive mechanical stress which perpetuates lung injury; a process referred to as ventilator-induced lung injury (VILI). The potential to induce VILI is more pronounced in the setting of pre-existing lung injury, such as ARDS, compared to the healthy lung [1].

While lung aeration is heterogeneous in the healthy lung due to innate variations in pulmonary structure [2], the ARDS lung has a higher degree of inhomogeneity [3, 4] which may facilitate the development of VILI. The importance of this is highlighted by the link between parenchymal inhomogeneity and mortality in patients with ARDS [3, 5]. While the implementation of protective ventilation strategies with low tidal volume was a landmark in improving clinical outcomes in ARDS patients [6], mortality remains unacceptably high [7, 8], and these low lung volumes may promote atelectasis [9, 10].

Despite the recognition of the important role of lung inhomogeneity in the causation of VILI, regional variations in the response to MV, and how this is altered by pre-existing lung injury, are poorly characterised. Knowledge in this area is important for the design of optimal MV strategies and therapeutic interventions to prevent/treat VILI. To date, few studies have attempted to link lung inflammation to regional deformation in the lungs of humans [11] and animal models [12, 13]. These studies have showed that neutrophilic activation and the expression of inflammatory markers (ICAM-1, CXCL8, IL-6, IL-1β, TNF-α) vary regionally in response to MV. However, we have recently shown that the association between the expression of these inflammatory cytokines and lung volumes is modified by prior treatment with lipopolysaccharide (LPS) [13]. In the healthy lung, regional tidal volume (Vt), but not end-expiratory lung volume (EEV), was positively associated with the regional expression of *IL6* [14], while, in the endotoxemic lung, there was a negative correlation between *IL-6* and EEV, but not Vt [13]. This implies that a priori selection of MV related lung injury markers may not be suitable for studying the interaction between MV and pre-existing lung injury. To achieve a greater depth of understanding, a broader approach is required.

Using samples collected from our previously published study [13], the aim of this study was to assess the regional transcriptome in the lung tissue of mice exposed to MV with or without pre-existing systemic inflammation induced by LPS. To our knowledge, transcriptomic analysis at the regional lung level, and linking this to the regional lung volume response, has not been reported previously. We hypothesised that region-specific transcriptomic changes would be associated with the regional lung volume response to MV and that this interaction would be modified by pre-existing inflammation. To achieve this, we profiled the transcriptome from 3 distinct lung regions using RNA sequencing (RNA-Seq) in mice that were mechanically ventilated for 2 h with or without prior exposure to intraperitoneal LPS. We then assessed the association between the dysregulated expression of biological pathway genes and regional lung volumes assessed by four-dimensional computed tomography (4DCT).

## Materials and methods

### Animal preparation and ventilation

The experimental protocol, which has been described previously [13], was approved by the Monash University and University of Tasmania Animal Ethics Committees (Ethics No: MARP-2014-137). Briefly, adult female BALB/c mice were injected intraperitoneally with 200 µL of 0.9% saline or 10 mg/kg lipopolysaccharide (LPS) in 200 µL of saline. Four hours after the injection, mice were either euthanased (by i.p. injection with 160 mg/kg sodium pentobarbitone) or surgically anaesthetised (ketamine 400 mg/kg: xylazine 20 mg/kg), tracheostomised and mechanically ventilated for 2 h (225 breaths/min, with a peak inspiratory pressure of 12 cmH_2_O and a PEEP of 2 cmH_2_O) prior to euthanasia. Thus, there were four experimental groups: Saline (Control), LPS, Saline/MV and LPS/MV (n = 6 mice per group). Mice in the ventilated groups were positioned upright, and 4DCT images were taken as described below.

### Lung imaging

As described previously [14], dynamic 4DCT images were segmented into 10 lung regions (four right lobes and six regions in the left lobe) to quantify regional tidal volume (Vt) and end-expiratory volume (EEV). To correct for the variation in regional lung size, we calculated specific Vt (regional Vt/total tidal volume of all voxels in the region) and specific EEV (regional EEV/total volume of all voxels in the region) for each region. Finally, we normalised the specific Vt and EEV to the corresponding region in the Saline control group to generate values for normalized Vt (nVT) and normalized EEV (nEEV) to facilitate between group comparisons.

### RNA isolation, RNA-Seq and qPCR

Total RNA was extracted from the 10 lung regions corresponding to the regional image segmentation described above. Amongst these, RNA samples of three regions (right superior lobe, R1; right post-caval lobe, R4; distal upper region of the left lobe, L2) were sent to the Australian Genome Research Facility for next generation RNA sequencing (RNA-Seq). For validation studies using qPCR, RNA samples were reverse transcribed, and the resulting cDNA samples were analysed for expression of seven VILI-related genes [*TNF-α*, *IL-1β*, *IL-6*, *Ccl2*, C-X-C motif chemokine ligand 2 (*Cxcl2*), myeloperoxidase (*Mpo*), and nuclear factor, erythroid 2-like 2 (*Nrf2*)] as reported previously [13].

### Regional inflammation (neutrophils)

In separate groups of mice, following euthanasia, lungs were harvested, embedded, sectioned and immunohistochemically stained using an anti-neutrophil [NIMP-R14] (5 ng/μL, Abcam, Australia) antibody and examined under light microscopy, as described previously [14]. The number of neutrophils per unit area were quantified in randomly selected images within each lung region from a single midline section that captured most of the lung regions.

### Differential expressed genes (DEGs) analysis

The raw gene read counts from the RNA-Seq analysis were converted into log_2_-counts per million, and were compared between each perturbation (MV, LPS, MV/LPS) using the EdgeR package [15]. Genes/transcripts with read counts ≤ 10 were excluded to reduce potential noise. *P*-values were adjusted for multiple testing using the Benjamini–Hochberg false discovery rate (FDR) method. A FDR < 0.05 was used to identify differentially expressed genes (DEGs). In the case of mice exposed to LPS (LPS and LPS/MV group), an additional criteria of log_2_FC > 1 (i.e. fold change > 2) was applied due to the high volume of differential expression. Data were visualized using the NetworkAnalyst platform [16].

### Bioinformatics analysis

The Gene Ontology (GO) enrichment and the Kyoto Encyclopedia of Genes and Genomes (KEGG) pathways were analysed using the DAVID bioinformatics resource [17]. Protein–protein interaction network analysis of DEGs was performed using the STRING database. A Benjamini-corrected *p* < 0.05 was used as the cut-off for all bioinformatics analyses.

### Statistical analysis

Data were analysed using the SPSS (version 20.0: SPSS Inc., Chicago, IL, USA) statistical package. Differences in regional lung volumes and neutrophils were analysed using two-way repeated measures ANOVA, with a Tukey significant difference test for post-hoc analysis. Aligned rank transformation was applied for non-parametric data. Principal component analysis (PCA) was conducted on identified gene sets. To enable inter-regional comparisons and establish the relationship between PCA scores and normalized lung volumes, individual PCA scores were normalized to the corresponding region of the Saline group; calculated as the difference in PCA (dPCA) scores relative to control PCA scores (PCA—mean of PCA in control region). Comparison of dPCA amongst multiple groups was analysed using One-Way ANOVA. The Pearson correlation index was calculated to determine the association among different continuous variables (such as between dPCA and lung volumes, between qPCR and RNA-Seq data), using linear regression analysis. Data are presented as mean (SD). Differences were considered statistically significant if *p* < 0.05.

## Results

### Regional lung volume responses

**Fig. 1:**
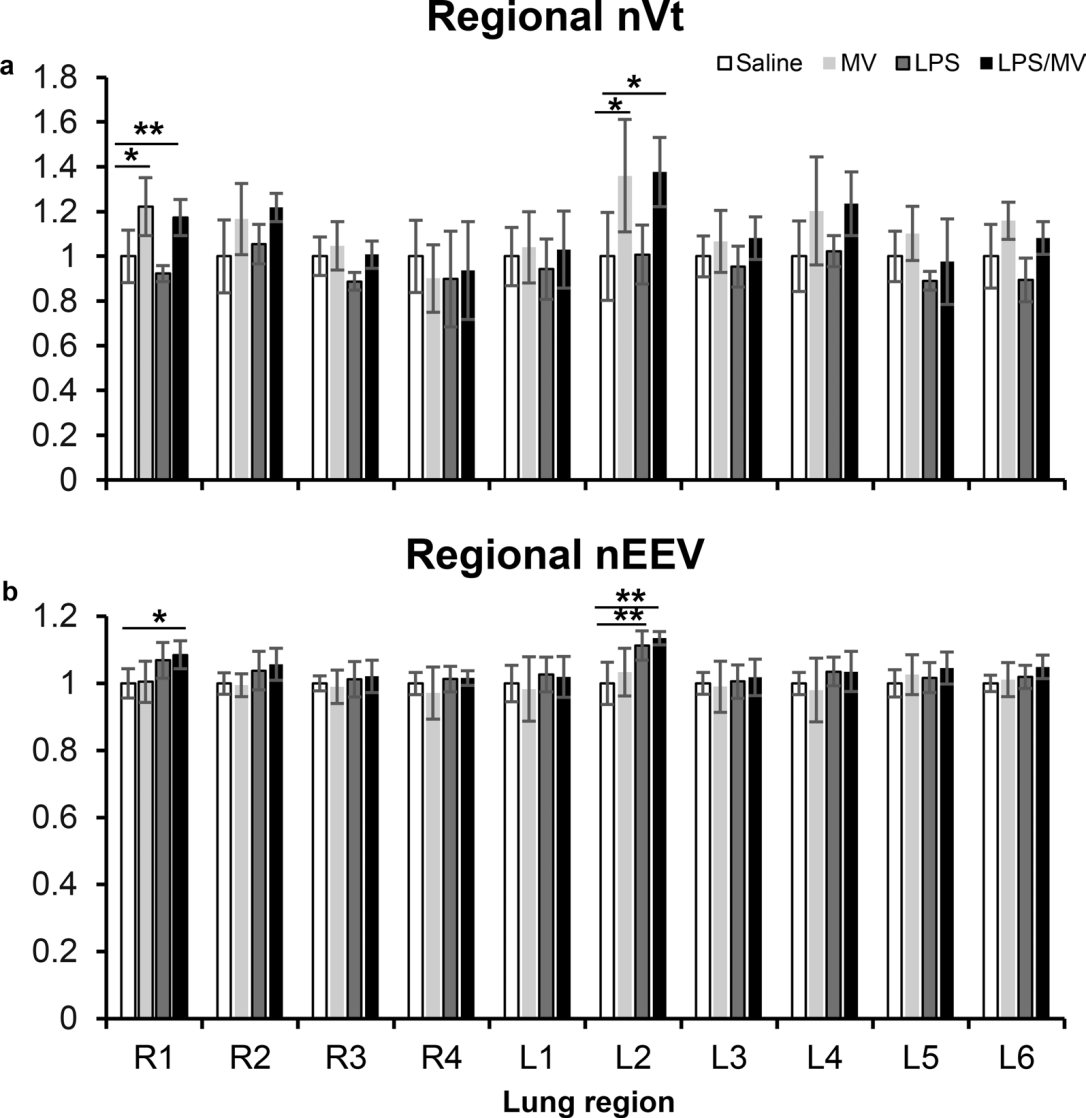
Regional lung volumes. Regional tidal volume (nVt) (**a**) and end-expiratory volume (nEEV) (**b**) normalized to baseline in the saline group for the LPS, MV and LPS/MV groups. Values are Mean (SD) with n = 6 per group. ^*^p ≤ 0.05, ^**^p < 0.01. LPS: lipopolysaccharide; MV: mechanical ventilation

As previously reported [13], there was inhomogeneity in Vt and EEV when normalized to the non-ventilated no-LPS controls which was mostly observed in the non-dependent lung regions (R1 and L2) (Fig. 1). There was a significant interaction between treatment and region for both nVt (*p* = 0.010) and nEEV (*p* = 0.015). The MV and LPS/MV groups had increased regional nVt after 2 h of ventilation in R1 and L2 (*p* < 0.05, Fig. 1a). There was no effect of LPS alone on regional nVt suggesting regional increases in nVt were due to a MV effect. The LPS/MV group had increased regional nEEV in R1 (*p* = 0.054) and L2 (*p* = 0.002), while non-ventilated LPS-treated mice also showed a significant increase in nEEV in L2 (*p* = 0.009) (Fig. 1b). In contrast, MV alone had no effect on regional nEEV (*p* > 0.05 for all regions; Fig. 1b) suggesting regional increases in nEEV were due to the effect of LPS.

### Identification of differentially expressed genes (DEGs)

The transcriptome sequencing led to identification of 14,879 genes with matches to known functions in the mouse genome. Similar to our previous report of regional heterogeneity in the protein profile [18], gene expression varied regionally in the control group. Specifically, 1015 genes in R1 or L2 had differential mRNA expression levels compared to those in R4 (FDR < 0.05), with 195 of these having a fold change > 2 (Additional file 3). This observation reinforces the necessity to normalize gene expression data to allow for spatial comparisons.

**Fig. 2:**
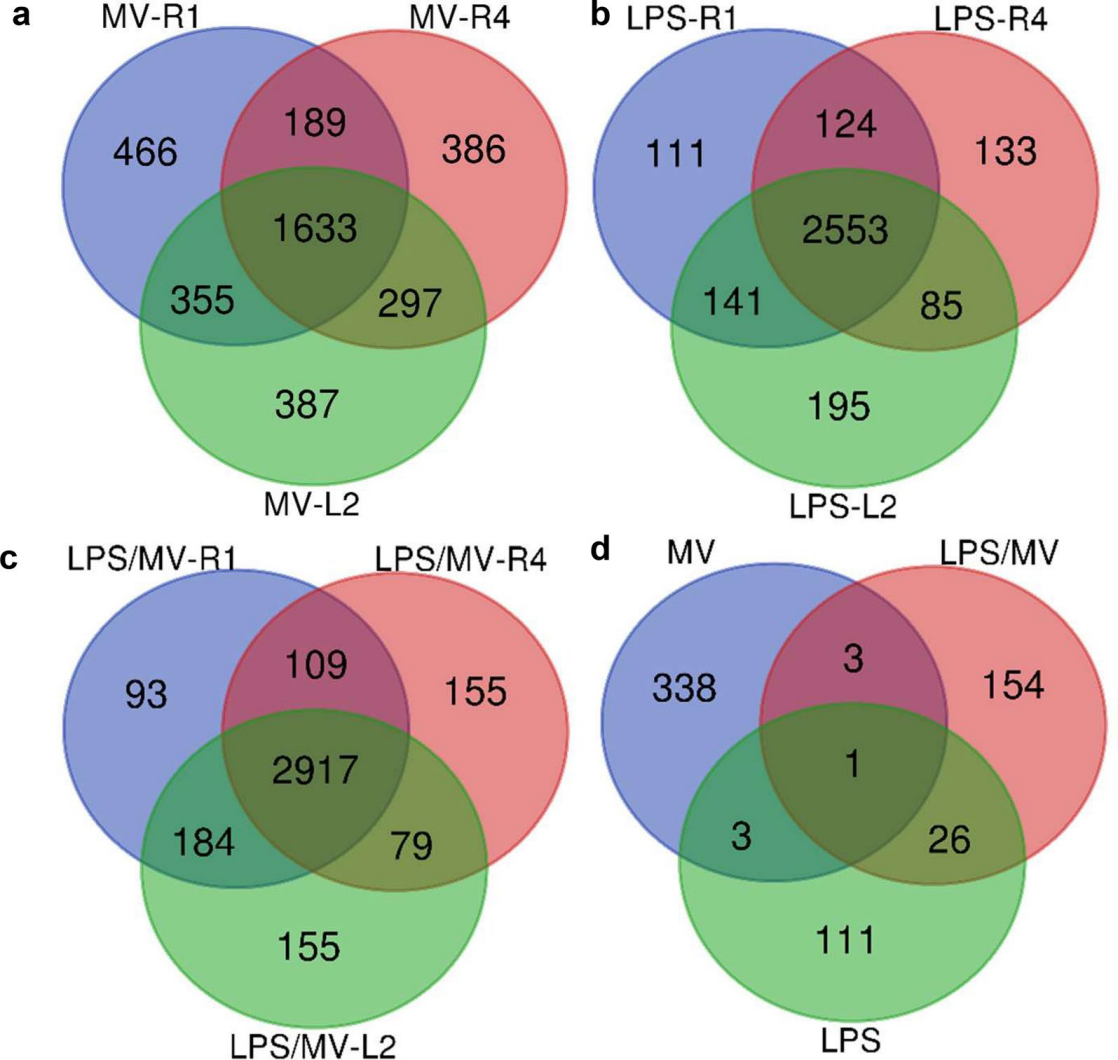
Venn diagram comparison of differentially expressed genes. The diagram depicts the number of overlapping and uniquely altered genes amongst the three regions (R1, R4 and L2) in three experimental conditions: MV (**a**), LPS (**b**) and LPS/MV (**c**). Panel **d** shows the comparison of altered genes unique to R1 and L2 in the three active experimental groups

Relative to the equivalent region in the control group, there were 2643, 2505 and 2672 DEGs in R1, R4 and L2 respectively in response to MV (Fig. 2a); 2929, 2895 and 2974 DEGs in R1, R4 and L2 respectively in response to LPS (Fig. 2b); and 3303, 3260 and 3335 DEGs in R1, R4 and L2 respectively in response to LPS/MV (Fig. 2c). Of these DEGs, there were 355 in response to MV (Fig. 2a), 141 in response to LPS (Fig. 2b) and 184 in response to LPS/MV (Fig. 2c) identified in R1 and L2, but not R4, with 26 of these shared in the LPS and LPS/MV groups and only 3 DEGs shared in the MV and LPS/MV groups (Fig. 2d). In further exploratory analyses, we focused on these genes and their possible link(s) with the regional lung volume heterogeneity we observed (Fig. 1).

### Functional characterization of DEGs

**Fig. 3:**
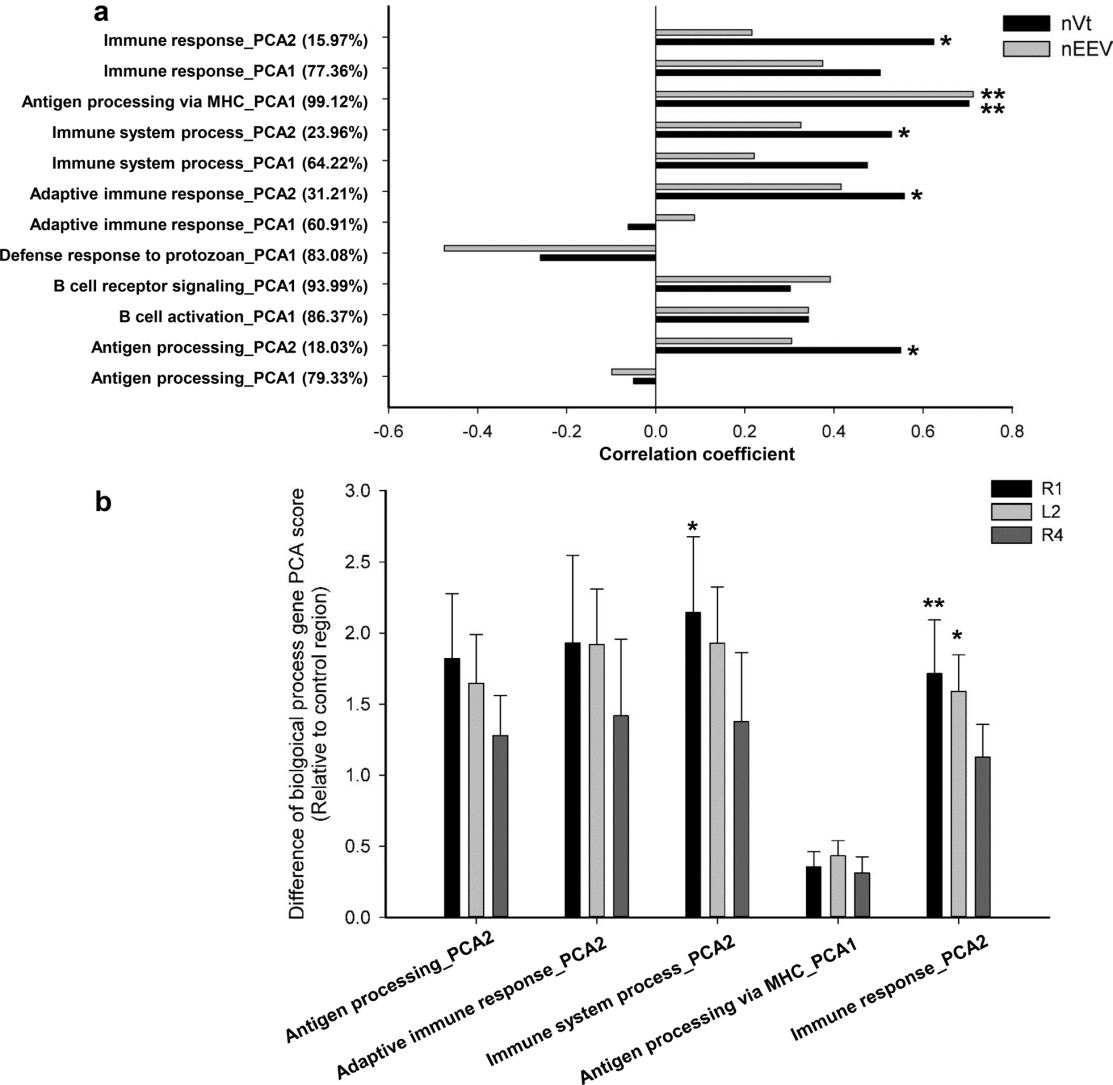
Functional gene clusters and the lung volume response to MV. Principal component analysis (PCA) was used to group genes in each functional category. The Pearson correlation coefficient was used measure the strength of the linear association between the generated PCA score for each category and lung volume (nVt and nEEV) in the MV group (n = 15) (**a**). PCA scores for the selected gene sets were compared amongst the three regions (R1, L2 and R4) after MV (**b**) (n = 6 per group; Values are mean + SD). ^*^ *p* < 0.05, ^**^ *p* < 0.01 (compared to R4 for graph B). nVt: normalized tidal volume; nEEV: normalized EEV

In analysing 345 region-specific DEGs (10 genes were excluded due to inconsistent expression changes in R1 and L2) in the MV group, functional annotation revealed 8 enriched and meaningful gene ontology (GO) terms composed of 42 up-regulated genes, all relating to immune responses such as B cell activation/signalling, defense responses and immune/antigen processing (Fig. 3a, Additional file 2: Table S1). In parallel, KEGG pathway analysis identified 13 enriched pathways associated with various autoimmune disorders (Additional file 2: Table S1).

In contrast, analysis (adjusted for FDR) of region-specific expression of 141 DEGs in the LPS group did not result in identification of any significant biological processes or KEGG pathways (Additional file 2: Table S2). Similarly, we did not find enriched biological processes using the 184 region-specific candidate genes in the LPS/MV group (Additional file 2: Table S3). Nevertheless, 13 clusters of genes were identified and, although these identified pathways did not reach statistical significance (adjusted *p* > 0.05), with the exception of non-small cell lung cancer pathway (*p* = 0.02) (Additional file 2: Table S3), they were consistent with gene clusters we identified in subsequent regional analyses (see below).

Given that the regional biological response to LPS and LPS/MV was subtle, we assessed the regional difference in pathway expression in the LPS and LPS/MV groups using the full set of DEGs for each region. We found that the majority of dysregulated pathways were shared by the three regions for both the LPS group (62 common pathways) and LPS/MV group (65 common pathways) (Additional file 1: Figure S1; details in Additional files 4 and 5). Almost all of these pathways and enriched GO terms were related to immune-inflammation responses (e.g. cytokine-cytokine receptor interaction, TNF signaling pathway, chemokine signaling pathway etc.; GO:0002376 ~ immune system process, GO:0006954 ~ inflammatory response, GO:0032496 ~ response to lipopolysaccharide etc.).

Of the region-specific pathways identified using this approach, 4 were identified in both the LPS and LPS/MV groups, including mmu05221: acute myeloid leukemia, mmu04725: cholinergic synapse, mmu04068: foxO signaling pathway and mmu04071: sphingolipid signaling pathway (Additional file 1: Figure S1). It is interesting to note that of 13 region-specific dysregulated pathways in the LPS/MV group, 12 of these were also identified in the previous analysis of 184 region-specific genes prior to correction for the FDR (Additional file 2: Table S3). Further comparison revealed that more genes in these pathways were dysregulated in R1 and L2, compared to R4, particularly in the LPS/MV group (Additional file 1: Figure S2).

### Association between gene expression and lung volumes

The identified gene sets in each pathway were grouped using PCA prior to correlating them with lung volumes. In the MV group, strong positive correlations were found between 5 biological processes (immune responses, antigen processing via MHC, adaptive immune responses, immune system processes, and antigen processing) and nVt (r > 0.5 for all correlations, Fig. 3a). Moreover, there was a significant association between antigen processing via MHC and nEEV (*p* < 0.01, Fig. 3a). Further analysis of these 5 biological pathways showed that the dPCA score for both R1 and L2 were significantly higher than those in R4 for immune system processes and immune responses, with the other biological pathways showing a similar trend (Fig. 3b).

**Fig. 4:**
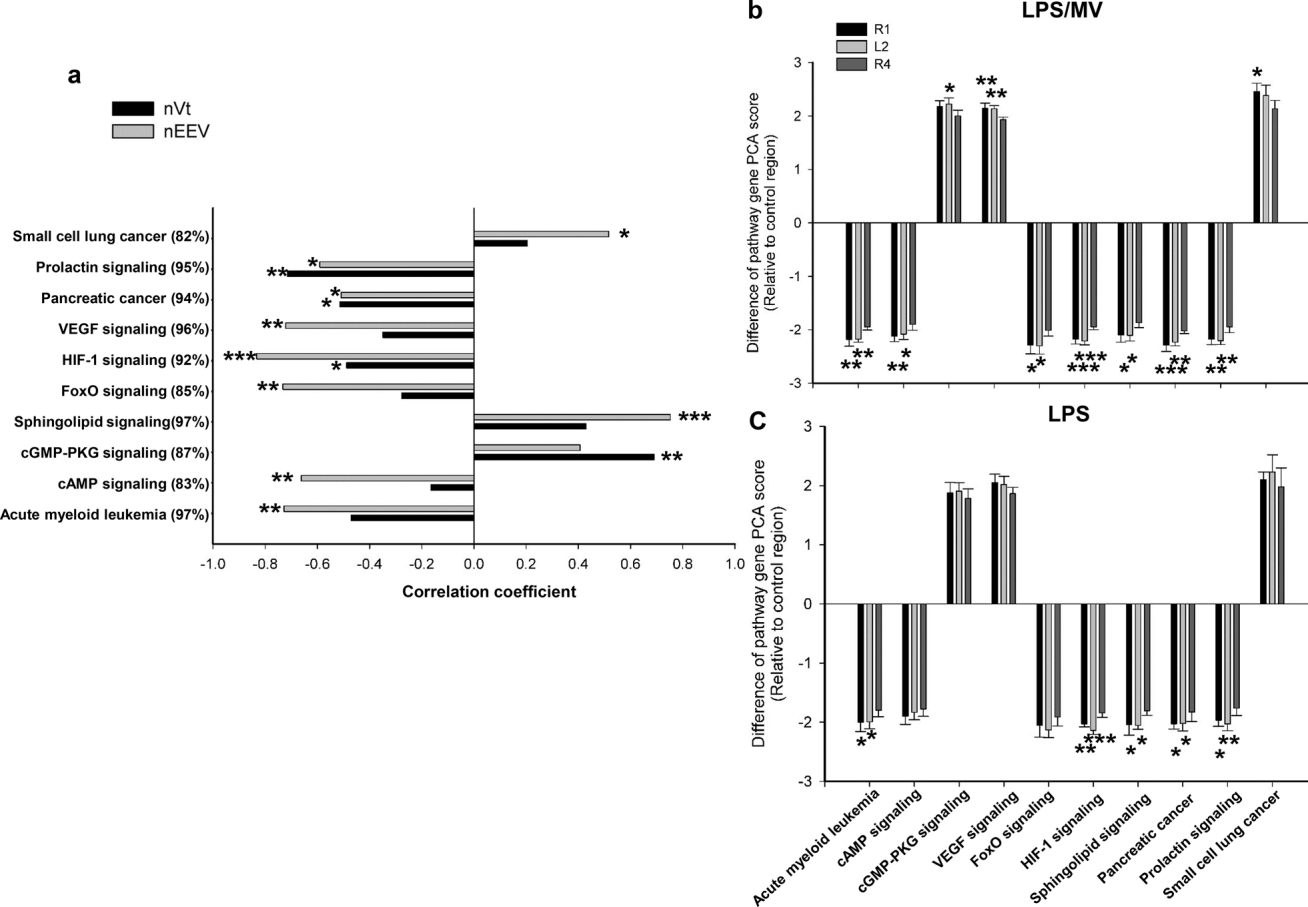
Functional gene clusters and the lung volume response to LPS/MV. Principal component analysis (PCA) was used to group genes in each functional category. The Pearson correlation coefficient was used to measure the strength of the linear association between the generated PCA score for each category and lung volume (nVt and nEEV) in the LPS/MV group (n = 17) (**a**). PCA scores for the selected gene sets were compared amongst the three regions (R1, L2 and R4) in the LPS/MV (**b**) and LPS (**c**) group (n = 6 for R1 and L2, n = 5 for R4; Values are mean + SD). ^*^ *p* < 0.05, ^**^ *p* < 0.01, ^***^p < 0.001 (compared to R4 for graph **b** & **c**). nVt: normalized tidal volume; nEEV: normalized EEV; MV: mechanical ventilation

However, these associations were not consistently observed in the LPS/MV group (Additional file 2: Table S4). In comparing the PCA scores of the three regions in the LPS and LPS/MV, we found that these sets of genes were increased after LPS exposure. As such, it is possible that the addition of MV did not further elevate the gene expression in the LPS treated groups (Additional file 2: Table S5). In the LPS/MV group, we assessed 10 pathways (Fig. 4). The expression of these pathways was significantly correlated with nVt or nEEV (Fig. 4a). All pathways, except for the cGMP-PKG signalling pathway, were significantly associated with nEEV, while 4 were associated with nVt. In line with this, the dPCA scores were significantly higher in R1 and L2 than those in the R4 region (Fig. 4b). Importantly, similar effects were observed for the LPS group (Fig. 4c).

**Fig. 5:**
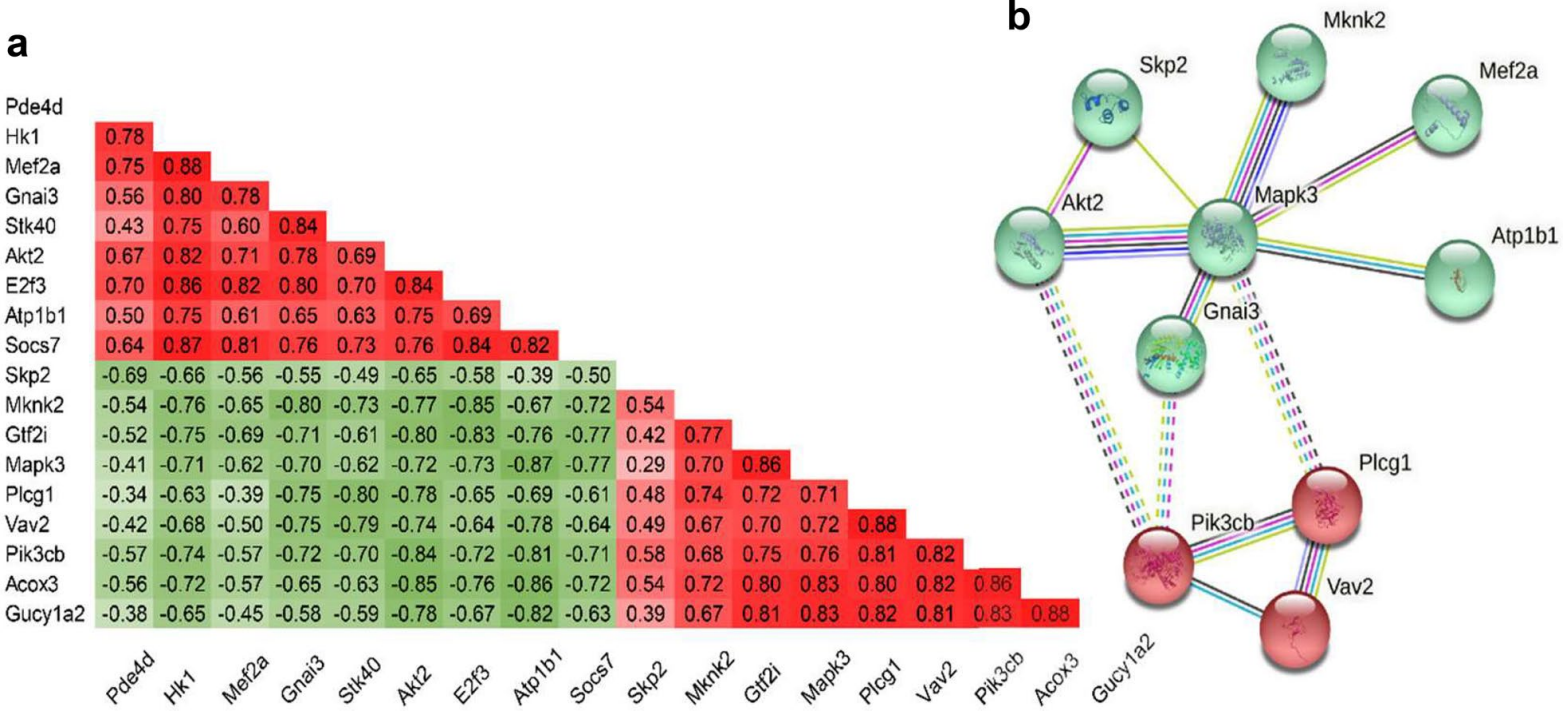
Correlation matrix and protein interaction network. Pearson correlation coefficient was calculated for the expression of specific pathways in the LPS/MV group (**a**). Green and red represent negative and positive associations, respectively. The colour intensity corresponds to the magnitude of the correlation coefficient. A protein–protein interaction regulatory network based on these genes was developed (**b**)

The 10 biological pathways were primarily composed of 18 genes (Additional file 2: Table S3). These key pathway genes were highly correlated with one another (Fig. 5a). Construction of a protein–protein interaction network using this subset of genes led to discovery of the two clusters, PI3K/Akt and MEK/ERK, in which 3 genes (Akt2, Mef2a and Atp1b10) were up-regulated and 5 were down-regulated (Fig. 5b).

### Confirmation of RNA-Seq data by RT-qPCR

To validate the sequencing results, the expression of seven genes, which we have previously determined using real-time qPCR [13], were correlated to the corresponding RNA-Seq data. Six genes (*TNF-α*, *IL-1β*, *IL-6*, *Ccl2*, *Cxcl2*, *Mpo*) exhibited very strong positive correlation between mRNA levels quantified by qPCR and RNA-Seq (r > 0.900, *p* < 0.001), while one (*Nrf2*) only showed a weak correlation (r = 0.379, *p* = 0.001) (Additional file 1: Figure S3). The latter observation is likely due to the between-group variance in *Nrf2* (< twofold) being lower than that in the other six genes (26–1306 fold). Nevertheless, these data confirmed the reliability of the RNA-Seq results obtained in this study.

### Neutrophil influx

Neither MV nor LPS alone induced significant infiltration of neutrophil cells (*p* > 0.05) (Additional file 1: Figure S4) into the lung. There was, however, a significant increase in numbers of neutrophil in response to LPS/MV, compared to the other three groups: Saline, MV and LPS (*p* < 0.05) (Additional file 1: Figure S4). We found no evidence of regional differences in this neutrophil influx (*p* = 0.187).

## Discussion

The aim of this study was to determine the association between regional gene expression and the response to mechanical ventilation (MV) with and without pre-existing lung injury. This work complements our previous studies by expanding the coverage of gene expression using a transcriptomic approach (RNA-Seq). Our data show that MV, despite being delivered at low tidal volume with PEEP, caused overstretch in the superior non-dependent lobes (R1 and L2) which was accompanied by up-regulation of genes related to immune responses. When combined with LPS-induced systemic inflammation, this caused an increase in EEV which was associated with dysregulated expression of a range of genes belonging to endothelial barrier related pathways. Our data unravelled different biological mechanisms associated with lung volume inhomogeneity during MV, with or without pre-existing lung injury, and highlight the complex network of pathways underpinning the development of lung pathology in this context.

As described previously, our imaging data show that low tidal volume ventilation induced regional over-stretch and, when combined with pre-existing systemic inflammation, increased regional EEVs [13, 14]. This is well aligned with “two-hit” concept that lungs with pre-existing injury are more susceptible to VILI [5], even with low tidal volume MV. However, the effect of protective MV strategies on the healthy lung (“one-hit”) is less clear [9]. Our data provide evidence supporting the direct effect of MV without pre-existing lung injury. This is somewhat in contradiction with our prior study whereby the same MV strategy in the context of intratracheal saline administration as a control had minimal effect on lung volumes [18], suggesting that intratracheal liquid aspiration may influence the MV response.

To determine whether this lung inhomogeneity was associated with the tissue response, we compared the regional gene expression profile, with a particular focus on common DEGs present in both R1 and L2, but absent in R4 (a region with no change in lung volumes in response to MV or LPS). Overall, our RNA-Seq transcript differential analysis under different experimental conditions, and across three lung regions, revealed several predicted responses as well as identification of novel pathways. These include (1) the greatest transcriptional response was in the LPS/MV group which is comparable to previous reports [19, 20], supporting the concept that gene transcription is significantly modulated by the interaction between MV and LPS; (2) while the number of DEGs for MV alone was comparatively low compared to the LPS response, our data also demonstrate that a protective ventilation strategy (in the absence of a primary pulmonary insult) induces a distinct transcriptional response in the lung [21], and; (3) most of region-specific DEGs in response to LPS/MV were unique to that group, suggesting an important role for the interaction between pre-existing lung injury and the response to MV in regulating the regional transcriptional response.

We subsequently performed bioinformatics analysis on the 345 region-specific DEGs in the MV group to gain insight into how MV contributes to the regional biological response in relation to exacerbation of lung volume inhomogeneity. These DEGs were mainly enriched in functions related to immune system processes, B cell activation, antigen processing and involving a variety of autoimmune disease related pathways. We further showed that up-regulation of these genes, particularly those related to immune responses, was associated with higher tidal stretch which was consistent with the between region analysis showing that the expression of these genes was higher in the regions exposed to high tidal stretch (e.g. R1 and L2), compared to those that were not (e.g. R4).

MV is thought to initiate subclinical VILI in the healthy lung which manifests as activation of proinflammatory transcriptional pathways [22], minor biochemical and histological changes [23]. It is likely that these changes sensitize the lung to further injury [9]. Human studies have shown that low tidal volume MV disrupts the Th1/Th2 balance in children without pre-existing lung pathology [24]. Similarly, animal studies have identified a role for activation of innate immunity in the pathogenesis of VILI using large tidal volume ventilation, while moderate tidal volume ventilation was found to augment the innate immune response and render the lung susceptible to the effects of systemic endotoxin [25]. Our data are consistent with this in that we have demonstrated that regional variations in the expression of immune response genes were linked with tidal stretch. Pre-exposure to LPS also up-regulated a similar suite of immune genes, however, subsequent MV does not appear to further increase the expression levels of these genes. This phenomenon may be explained by a homeostatic mechanism that prevents excessive activation of innate immune responses to a secondary stimulus [26].

The ARDS lung is usually characterized by a high degree of inhomogeneity which is associated with disease severity and mortality [3]. Indeed, the same lung may show a wide spectrum of aeration, ranging from complete atelectasis to hyperinflation. This inhomogeneity is normally caused by the presence of lung edema [4] and its maldistribution in relation to superimposed gravitational pressure. This is consistent with our observation in that R1 and L2 became hyperinflated at end-expiration after LPS exposure compared to the control. Our transcriptomic analysis identified a large number of molecular pathways related to inflammation and immune responses that were dysregulated following LPS administration with the majority of these (65 in LPS/MV; 62 in LPS) being consistently disrupted across L1, L2 and R4.

While the regional gene response in the LPS and LPS/MV groups was subtle compared to the overall effect of LPS and MV, we were able to identify 13 dysregulated region-specific pathways in the LPS/MV group, which were associated with alterations in lung volumes (Vt and EEV). Of these, 9 were unique to the LPS/MV group suggesting that they are specific to the interaction between pre-existing lung injury and the response to MV. It is interesting to note that most of these pathways are linked to MV-induced endothelial barrier dysfunction, while three are involved in cell growth and differentiation in maintaining endothelial integrity. There is evidence to suggest that loss of endothelial barrier function plays a primary role in the pathogenesis of VILI and ARDS. For example, high Vt-induced endothelial barrier dysfunction has been attributed to an increase in NO/sGC-derived cGMP and subsequent decrease in cAMP expression [27]. Similarly, the roles of FOXO and VEGF in endothelial cell signaling are well-established in the context of endothelial barrier function and lung injury [28]. Sphingolipid and sphingolipid metabolizing enzyme(s) have been linked to epithelial and endothelial permeability and lung apoptosis [29]. Our data showed that 3 pathways (small cell lung cancer, cGMP-PKG signaling and sphingolipid signaling) in the LPS/MV group were positively correlated with lung volumes while 7 were negatively associated with the lung parameters, which is in alignment with the known roles of these pathways in endothelial barrier dysfunction. Using the gene clusters linked to these pathways, we constructed a protein–protein interaction network which led to the identification of PI3K/Akt and MEK/ERK signalling clusters. On this basis, we propose that PI3K/Akt and MEK/ERK signalling is likely to play a central role in the development of regional lung injury in response to the interaction between LPS exposure and MV.

Although the accuracy and reliability of our RNA-Seq data was well validated by an independent assay (qPCR), there are several limitations in the transcriptomic data we generated that should be acknowledged. First, our interpretation of changes in pathways / biological processes was based on gene expression and bioinformatics analysis and, while novel, the findings need to be confirmed at a functional level (e.g. protein abundance), or, ideally, by mechanistic studies. This is particularly important considering that a number of pathways were altered (some up-regulated, some down-regulated). Second, our data showed that LPS/MV increased the influx of neutrophils which may have impacted on the expression of the pathways that we have identified. An approach such as single cell RNA-Seq analysis is needed to remove the confounding effects of infiltrating inflammatory cells which may alter the gene expression profile [30] to reveal the VILI-related gene signatures that are linked to heterogeneity of responses in the lung tissue itself. However, it should be noted that the influx of neutrophils was only evident in the LPS/MV group and we found no evidence of regional variation in this response. This suggests that the MV-specific responses, and the region-specific responses, are unlikely to have been influenced by localised changes in inflammation.

## Conclusion

Despite these limitations, we have identified multiple gene clusters that were associated with the regional lung volume response to MV with or without prior exposure to LPS. The biological processes associated with lung inhomogeneity during MV, and MV in the presence of LPS, differed in that MV primarily induced up-regulation of regional immune response genes while LPS/MV disrupted PI3K/Akt and MEK/ERK signalling which interact with multiple molecular pathways. In the case of MV, these responses were primarily associated with regional tidal stretch. Conversely, regional variations in the LPS/MV group were primarily linked to end-expiratory gas trapping. Thus, our study provides novel molecular insights into the mechanisms driving lung inhomogeneity during VILI, although identification of the exact causal and regulatory mechanisms requires further investigation.

## Supplementary Information

**Additional file 1**: **Figure S1**. Regional pathways identification. **Figure S2**. Regional comparison of the selected pathways. **Figure S3**. Concordance in gene expression intensities between RNA-Seq and qPCR. **Figure S4**. Comparison of regional neutrophil numbers.

**Additional file 2**: **Table S1**. Bioinformatic analysis on 345 dysregulated genes in R1 and L2 after 2 h MV. **Table S2**. Bioinformatic analysis on 141 dysregulated genes in R1 and L2 after LPS. **Table S3.** Bioinformatic analysis on 184 dysregulated genes in R1 and L2 after LPS/MV. **Table S4**. Correlation between MV induced region-specific gene expression and lung volumes in LPS/MV group. **Table S5**. Comparison of MV induced region-specific gene expression in the three regions under experimental conditions.

**Additional file 3**: Supplementary data for regional difference in Saline group.

**Additional file 4**: Supplementary data for bioinformatic analysis of three lung regions after LPS/MV.

**Additional file 5**: Supplementary data for bioinformatic analysis of three lung regions after LPS.

## Data Availability

The datasets used and/or analysed during the current study are available from the corresponding author upon reasonable request.

## Abbreviations

MV: Mechanical ventilation
ARDS: Acute respiratory distress syndrome
VILI: Ventilator-induced lung injury
LPS: Lipopolysaccharide
Vt: Tidal volume
EEV: End-expiratory lung volume
RNA-Seq: RNA sequencing
4DCT: Four-dimensional computed tomography
nVT: Normalized Vt
nEEV: Normalized EEV
*Cxcl2*: C-X-C motif chemokine ligand 2
*Mpo*: Myeloperoxidase
*Nrf2*: Nuclear factor, erythroid 2-like 2
DEGs: Differential expressed genes
FDR: False discovery rate
PCA: Principal component analysis
dPCA: Difference in PCA (dPCA) scores relative to control PCA scores
