# Supplementary material for: The association between regional transcriptome profiles and lung volumes in response to mechanical ventilation and lung injury
Source: Respir Res. 2022 Feb 19;23:35. doi: 10.1186/s12931-022-01958-2 (PMC8857787; doi:10.1186/s12931-022-01958-2)
Supplement: Supplementary file 1 — Additional file 1: Figure S1. Regional pathways identification. Figure S2. Regional comparison of the selected pathways. Figure S3. Concordance in gene expression intensities between RNA-Seq and qPCR. Figure S4. Comparison of regional neutrophil numbers. [file 12931_2022_1958_MOESM1_ESM.docx]

**Title:** The association between regional transcriptome profiles and lung volumes in response to mechanical ventilation and lung injury

**Running head:** Regional lung transcriptome and mechanical ventilation

Yong Song^1^, Seiha Yen^1^, Melissa Preissner^2^, Ellen Bennett^1^, Stephen Dubsky^2^, Andreas Fouras^3^, Peter A. Dargaville^1^, Graeme R. Zosky^1,4 *^

*^1^Menzies Institute for Medical Research, College of Health and Medicine, University of Tasmania, Hobart, Tasmania, Australia.*

*^2^Department of Mechanical and Aerospace Engineering, Monash University, Melbourne, Victoria, Australia.*

*^3^4Dx Limited, Melbourne, Victoria, Australia.*

*^4^School of Medicine, College of Health and Medicine, University of Tasmania, Hobart, Tasmania, Australia.*

^*^Address correspondence to:

*Graeme R. Zosky (PhD); Address:* *Menzies Institute for Medical Research, College of Health and Medicine, University of Tasmania, Hobart, Tasmania, Australia; Ph: +61 3 6226 6921;* *Email: Graeme.Zosky@utas.edu.au*


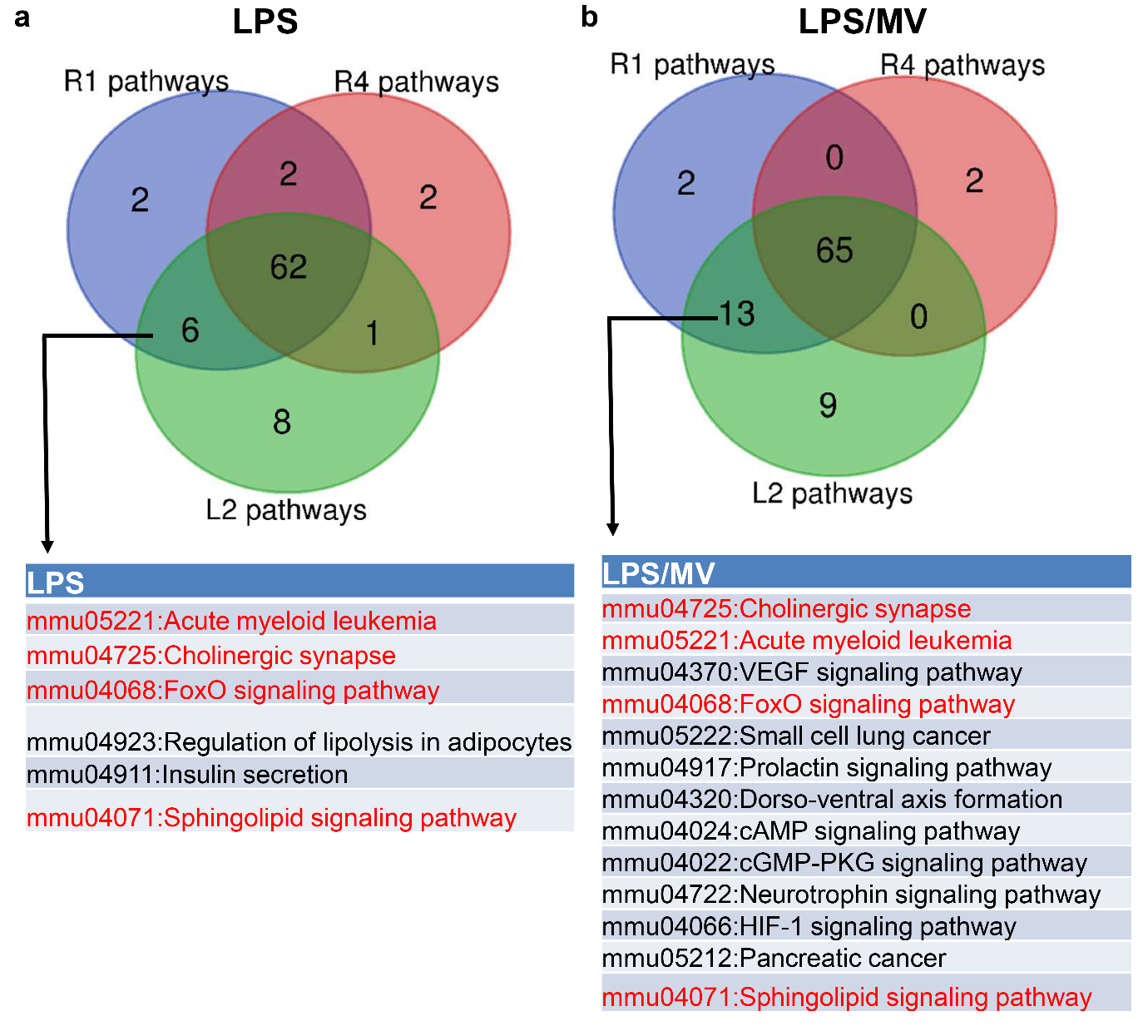


**Figure S1 – Regional pathways identification:** The Venn diagram depicts the number of overlapping and uniquely altered pathways amongst the three regions (R1, R4 and L2) in two experimental conditions: LPS (a) and LPS/MV (b). The dysregulated pathways commonly presented in R1 and L2 are shown under the corresponding Venn diagram, and highlighted with red for both LPS and LPS/MV injuries.


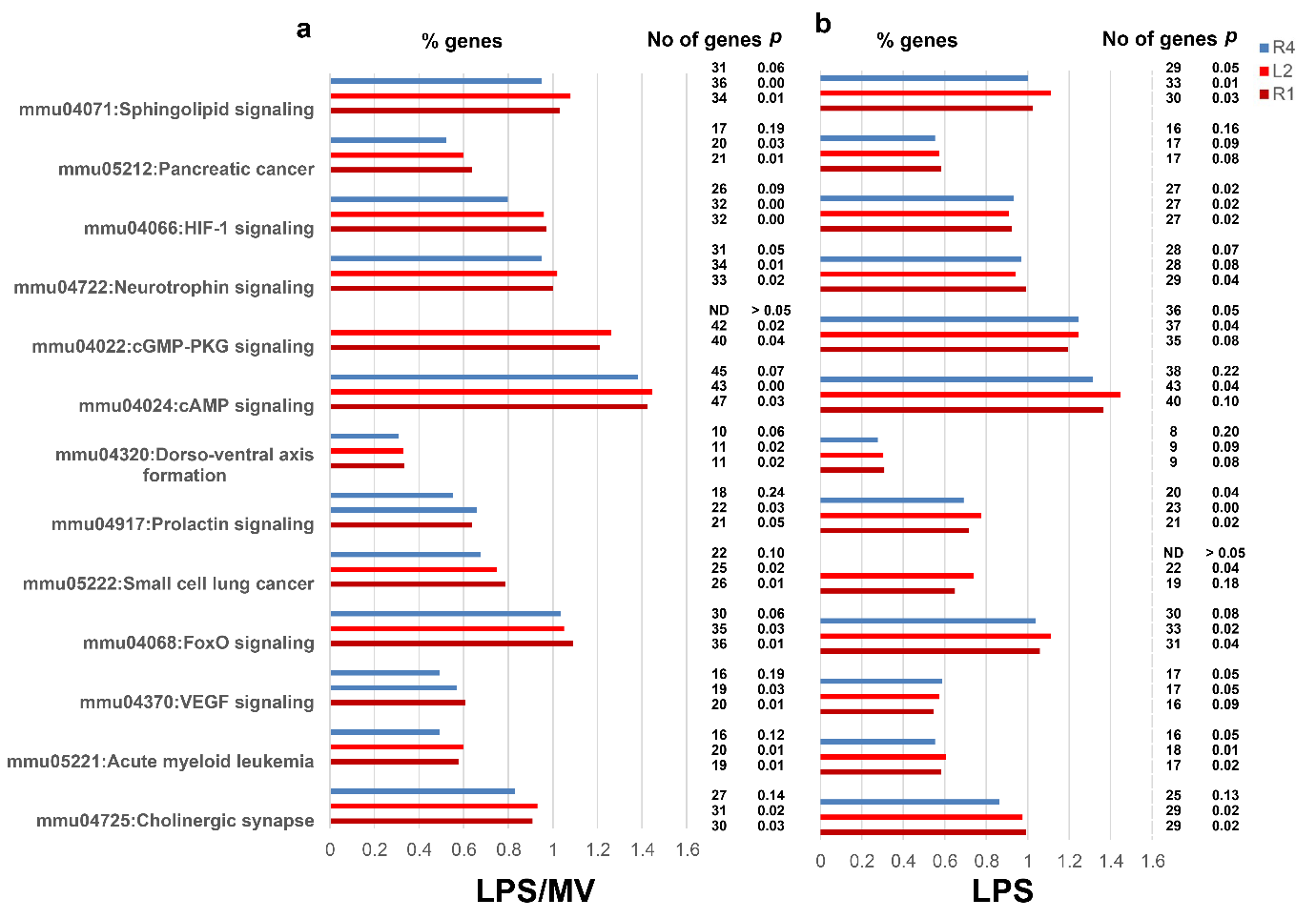


**Figure S2 – Regional comparison of the selected pathways:** The selected 13 unique pathways in R1 and L2 dysregulated in LPS/MV were summarized and compared in the three regions in two experimental conditions: LPS/MV (a) and LPS (b). Benjamini *p* value is shown.


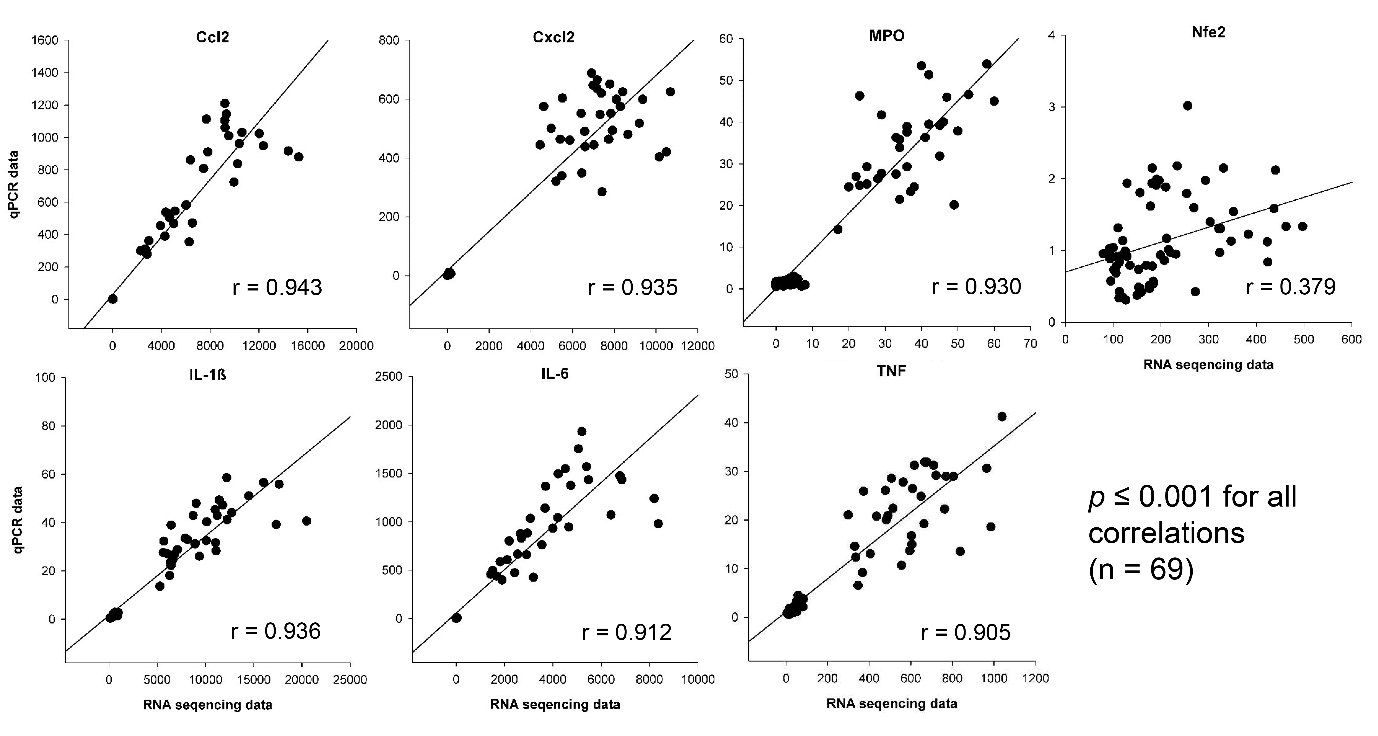


**Figure S3 – Concordance in gene expression intensities between RNA-Seq and qPCR:** Correlation of 7 selected genes expression between normalized RT-qPCR fold change values and raw RNA-Seq expression values was analysed by calculating Pearson correlation coefficient (r).


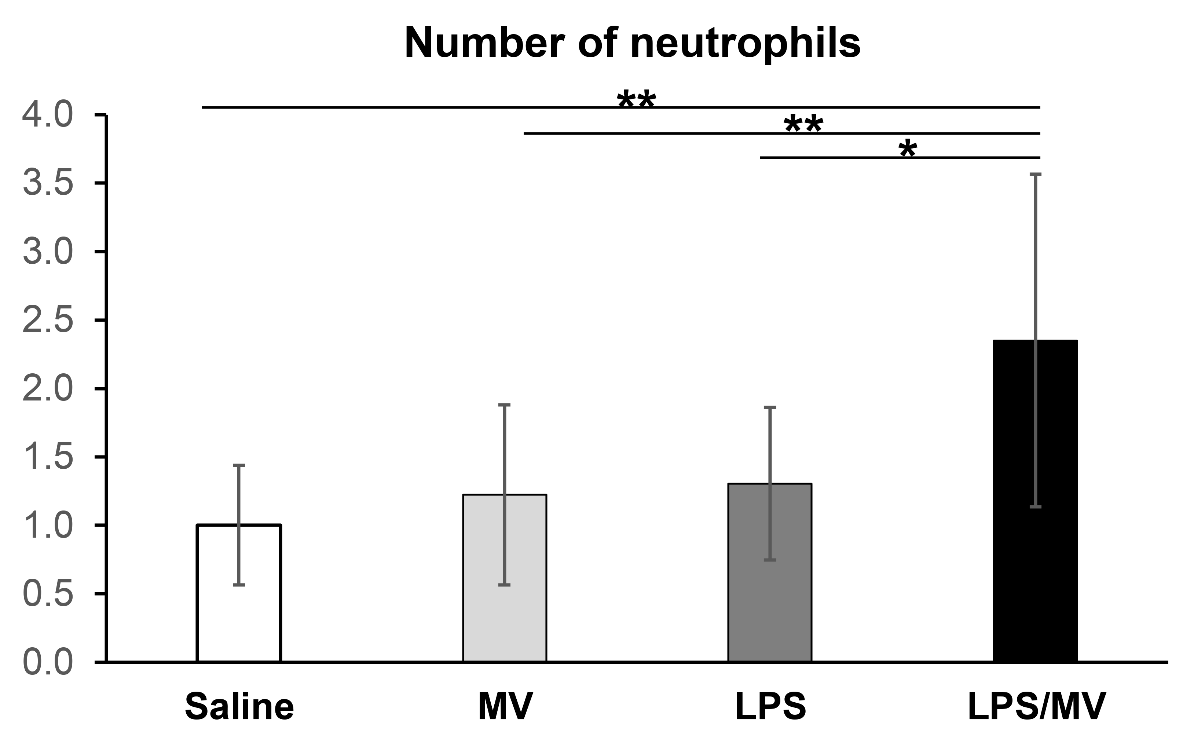


**Figure S4 – Comparison of regional neutrophil numbers:** The graph shows overall comparison of neutrophil numbers for the four experimental groups: Saline (n=48), MV (n=56), LPS (n=48) and LPS/MV (n=64). Values are Mean (SD). Overall *p* = 0.007. ^*^*p* < 0.05, ^**^*p* < 0.01.
