# Supplementary material for: The association between regional transcriptome profiles and lung volumes in response to mechanical ventilation and lung injury
Source: Respir Res. 2022 Feb 19;23:35. doi: 10.1186/s12931-022-01958-2 (PMC8857787; doi:10.1186/s12931-022-01958-2)
Supplement: Supplementary file 2 — Additional file 2: Table S1. Bioinformatic analysis on 345 dysregulated genes in R1 and L2 after 2 h MV. Table S2. Bioinformatic analysis on 141 dysregulated genes in R1 and L2 after LPS. Table S3. Bioinformatic analysis on 184 dysregulated genes in R1 and L2 after LPS/MV. Table S4. Correlation between MV induced region-specific gene expression and lung volumes in LPS/MV group. Table S5. Comparison of MV induced region-specific gene expression in the three regions under experimental conditions. [file 12931_2022_1958_MOESM2_ESM.docx]

**Title:** The association between regional transcriptome profiles and lung volumes in response to mechanical ventilation and lung injury

**Running head:** Regional lung transcriptome and mechanical ventilation

Yong Song^1^, Seiha Yen^1^, Melissa Preissner^2^, Ellen Bennett^1^, Stephen Dubsky^2^, Andreas Fouras^3^, Peter A. Dargaville^1^, Graeme R. Zosky^1,4 *^

*^1^Menzies Institute for Medical Research, College of Health and Medicine, University of Tasmania, Hobart, Tasmania, Australia.*

*^2^Department of Mechanical and Aerospace Engineering, Monash University, Melbourne, Victoria, Australia.*

*^3^4Dx Limited, Melbourne, Victoria, Australia.*

*^4^School of Medicine, College of Health and Medicine, University of Tasmania, Hobart, Tasmania, Australia.*

^*^Address correspondence to:

*Graeme R. Zosky (PhD); Address:* *Menzies Institute for Medical Research, College of Health and Medicine, University of Tasmania, Hobart, Tasmania, Australia; Ph: +61 3 6226 6921;* *Email: Graeme.Zosky@utas.edu.au*

**Table S1: Bioinformatic analysis on 345 dysregulated genes in R1 and L2 after 2 h MV.**

| **Gene Ontology Term** | **No.** | **%** | **Genes** | **Fold Enrichment** | ***p*** | **Adjusted *p*** |
| --- | --- | --- | --- | --- | --- | --- |
| GO:0002376~immune system process | 22 | 11.22 | ITK, CD40, H2-EB1, SP110, PRKCB, H2-K1, RNF19B, TAP1, TNFRSF13C, SERPINA3G, CD79B, CD79A, PSTPIP1, AIM2, CASP4, BTLA, PTK2B, PGLYRP1, B2M, H2-D1, H2-Q10, TLR13 | 6.26 | 4.79E-11 | 4.97E-08 |
| GO:0042832~defense response to protozoan | 7 | 3.57 | GBP6, CD40, GBP10, IRF4, BCL3, IRF8, CD37 | 25.42 | 2.61E-07 | 1.35E-04 |
| GO:0002250~adaptive immune response | 10 | 5.10 | CD79B, CD79A, ITK, PRKCB, BTLA, RNF19B, TAP1, PTK2B, TNFRSF13C, SERPINA3G | 7.84 | 5.25E-06 | 0.00 |
| GO:0042113~B cell activation | 6 | 3.06 | CD79A, CD40, BANK1, PRKCB, BLNK, IKZF3 | 22.54 | 5.92E-06 | 0.00 |
| GO:0019882~antigen processing and presentation | 6 | 3.06 | H2-EB1, H2-K1, GM11127, H2-OA, H2-D1, RELB | 12.10 | 1.31E-04 | 0.02 |
| GO:0050853~B cell receptor signaling pathway | 6 | 3.06 | CD79B, BLK, CD79A, PRKCB, CD19, RFTN1 | 11.88 | 1.43E-04 | 0.02 |
| GO:0006955~immune response | 11 | 5.61 | ENDOU, H2-EB1, CCL5, H2-K1, BLNK, CD28, IRF8, H2-OA, TNFRSF4, B2M, H2-D1 | 4.41 | 2.03E-04 | 0.03 |
| GO:0002474~antigen processing and presentation of peptide antigen via MHC class I | 5 | 2.55 | H2-K1, H2-M3, B2M, H2-D1, H2-Q10 | 15.13 | 3.14E-04 | 0.04 |
|  |  |  |  |  |  |  |
| **KEGG pathway** |  |  |  |  |  |  |
| mmu05340:Primary immunodeficiency | 7 | 3.57 | CD79A, CD40, CD19, RFXAP, BLNK, TAP1, TNFRSF13C | 18.63 | 1.49E-06 | 1.33E-04 |
| mmu05330:Allograft rejection | 8 | 4.08 | CD40, H2-EB1, H2-K1, H2-M3, CD28, H2-OA, H2-D1, H2-Q10 | 12.93 | 2.17E-06 | 1.33E-04 |
| mmu04612:Antigen processing and presentation | 9 | 4.59 | H2-EB1, H2-K1, RFXAP, H2-M3, TAP1, H2-OA, B2M, H2-D1, H2-Q10 | 9.93 | 2.67E-06 | 1.33E-04 |
| mmu05320:Autoimmune thyroid disease | 8 | 4.08 | CD40, H2-EB1, H2-K1, H2-M3, CD28, H2-OA, H2-D1, H2-Q10 | 10.20 | 1.09E-05 | 4.06E-04 |
| mmu05332:Graft-versus-host disease | 7 | 3.57 | H2-EB1, H2-K1, H2-M3, CD28, H2-OA, H2-D1, H2-Q10 | 12.18 | 1.93E-05 | 5.49E-04 |
| mmu05416:Viral myocarditis | 8 | 4.08 | CD40, H2-EB1, H2-K1, H2-M3, CD28, H2-OA, H2-D1, H2-Q10 | 9.16 | 2.21E-05 | 5.49E-04 |
| mmu04940:Type I diabetes mellitus | 7 | 3.57 | H2-EB1, H2-K1, H2-M3, CD28, H2-OA, H2-D1, H2-Q10 | 10.22 | 5.35E-05 | 0.001 |
| mmu05169:Epstein-Barr virus infection | 9 | 4.59 | CD40, CD19, H2-K1, H2-M3, NFKBIE, H2-D1, H2-Q10, RELB, JAK1 | 5.99 | 1.10E-04 | 0.00 |
| mmu04514:Cell adhesion molecules (CAMs) | 9 | 4.59 | CD40, H2-EB1, H2-K1, H2-M3, CD28, H2-OA, LRRC4C, H2-D1, H2-Q10 | 5.03 | 3.65E-04 | 0.01 |
| mmu05168:Herpes simplex infection | 10 | 5.10 | H2-EB1, DAXX, CCL5, H2-K1, H2-M3, TAP1, H2-OA, H2-D1, H2-Q10, JAK1 | 4.35 | 4.13E-04 | 0.01 |
| mmu04672:Intestinal immune network for IgA production | 5 | 2.55 | CD40, H2-EB1, CD28, TNFRSF13C, H2-OA | 10.77 | 0.001 | 0.02 |
| mmu04145:Phagosome | 8 | 4.08 | H2-EB1, TUBB2B, H2-K1, H2-M3, TAP1, H2-OA, H2-D1, H2-Q10 | 4.23 | 0.003 | 0.03 |
| mmu05166:HTLV-I infection | 10 | 5.10 | CD40, H2-EB1, H2-K1, H2-M3, TNFRSF13C, H2-OA, H2-D1, H2-Q10, RELB, JAK1 | 3.28 | 0.003 | 0.04 |

No: number of genes; %: gene percentage. Adjusted *p*: Benjamini-Hochberg *p* value.

**Table S2: Bioinformatic analysis on 141 dysregulated genes in R1 and L2 after LPS.**

| **Gene Ontology Term** | **No.** | **%** | **Genes** | **Fold Enrichment** | ***p*** | **Adjusted *p*** |
| --- | --- | --- | --- | --- | --- | --- |
| GO:0045429~positive regulation of nitric oxide biosynthetic process | 4 | 2.84 | AKT2, ULBP1, CLU, ASS1 | 14.22 | 0.00 | 1 |
| GO:0043065~positive regulation of apoptotic process | 8 | 5.67 | NGFR, SMPD2, RXRA, USP27X, UBD, DAB2IP, CTLA4, CLU | 3.82 | 0.00 | 1 |
| GO:0040008~regulation of growth | 4 | 2.84 | CCDC85B, DAB2IP, KCTD11, TSPYL5 | 9.28 | 0.01 | 1 |
| GO:0006470~protein dephosphorylation | 5 | 3.55 | DUSP22, PTPRR, DUSP3, FBXW11, TAB1 | 5.80 | 0.01 | 1 |
| GO:1901216~positive regulation of neuron death | 3 | 2.13 | NGFR, TRP53BP2, CLU | 17.78 | 0.01 | 1 |
| GO:0001701~in utero embryonic development | 6 | 4.26 | SERPINA1B, PTPRR, RXRA, DUSP3, TAB1, MTSS1 | 3.21 | 0.04 | 1 |
| GO:0006355~regulation of transcription, DNA-templated | 22 | 15.60 | HDAC4, USP13, ZFP667, USP27X, KDM1B, GTF3A, ZBTB1, GATA5, 2810021J22RIK, ZFP827, HDAC7, TOX3, ZFP322A, FOXS1, RXRA, CCDC85B, XRN2, LEO1, RCOR3, ZFP9, PHF19, ZIK1 | 1.54 | 0.04 | 1 |
| GO:0000185~activation of MAPKKK activity | 2 | 1.42 | DAB2IP, TAB1 | 40.00 | 0.05 | 1 |
|  |  |  |  |  |  |  |
| **KEGG pathway** |  |  |  |  |  |  |
| mmu04725:Cholinergic synapse | 5 | 3.55 | ACHE, AKT2, CAMK2A, KCNQ4, ADCY6 | 8.10 | 0.00 | 0.36 |
| mmu04611:Platelet activation | 5 | 3.55 | P2RX1, AKT2, GP5, ADCY6, PTGS1 | 6.99 | 0.00 | 0.36 |
| mmu00564:Glycerophospholipid metabolism | 4 | 2.84 | ACHE, PHOSPHO1, LPCAT2, AGPAT4 | 7.79 | 0.01 | 0.65 |
| mmu04114:Oocyte meiosis | 4 | 2.84 | FBXW11, CAMK2A, ADCY6, CPEB4 | 6.54 | 0.02 | 0.78 |
| mmu04923:Regulation of lipolysis in adipocytes | 3 | 2.13 | AKT2, ADCY6, PTGS1 | 9.64 | 0.04 | 1 |
| mmu04010:MAPK signaling pathway | 5 | 3.55 | PTPRR, DUSP3, AKT2, TAB1, MAP3K6 | 3.65 | 0.04 | 1 |

No: number of genes; %: gene percentage. Adjusted *p*: Benjamini-Hochberg *p* value.

**Table S3: Bioinformatic analysis on 184 dysregulated genes in R1 and L2 after LPS/MV.**

| **Gene Ontology Term** | **No.** | **%** | **Genes** | **Fold Enrichment** | ***p*** | **Adjusted *p*** |
| --- | --- | --- | --- | --- | --- | --- |
| GO:0050821~protein stabilization | 7 | 3.80 | USP13, TESC, PEX6, STK4, ATP1B1, CLU, SMAD7 | 6.30 | 0.00 | 0.39 |
| GO:0008284~positive regulation of cell proliferation | 14 | 7.61 | TPD52, WNT7B, HILPDA, LRP5, VEGFC, FOXM1, CLU, FGF7, FABP4, MYCN, CLCF1, ID2, E2F3, TRPM4 | 2.97 | 0.00 | 0.39 |
| GO:0001525~angiogenesis | 9 | 4.89 | ACVRL1, WNT7B, LEPR, COL8A1, VEGFC, CSPG4, PNPLA6, EPHB4, VAV2 | 4.34 | 0.00 | 0.39 |
| GO:0035556~intracellular signal transduction | 11 | 5.98 | GUCY1A2, DEPTOR, AKT2, MKNK2, CSPG4, PLCG1, STK4, SOCS7, MAPK3, VAV2, SMAD7 | 3.17 | 0.00 | 0.64 |
| GO:0045766~positive regulation of angiogenesis | 6 | 3.26 | ACVRL1, CYSLTR1, CMA1, VEGFC, PLCG1, ADD1 | 5.71 | 0.00 | 0.82 |
|  |  |  |  |  |  |  |
| **KEGG pathway** |  |  |  |  |  |  |
| mmu05223:Non-small cell lung cancer | 6 | 3.26 | AKT2, E2F3, PIK3CB, PLCG1, STK4, MAPK3 | 11.77 | 0.00 | 0.02 |
| mmu04024:cAMP signaling pathway | 8 | 4.35 | PDE4D, AKT2, GNAI3, PIK3CB, ACOX3, ATP1B1, MAPK3, VAV2 | 4.46 | 0.00 | 0.06 |
| mmu04066:HIF-1 signaling pathway | 6 | 3.26 | AKT2, MKNK2, PIK3CB, PLCG1, MAPK3, HK1 | 6.46 | 0.00 | 0.06 |
| mmu04022:cGMP-PKG signaling pathway | 7 | 3.80 | MEF2A, GUCY1A2, AKT2, GNAI3, ATP1B1, MAPK3, GTF2I | 4.72 | 0.00 | 0.06 |
| mmu04370:VEGF signaling pathway | 4 | 2.17 | AKT2, PIK3CB, PLCG1, MAPK3 | 7.32 | 0.02 | 0.14 |
| mmu05212:Pancreatic cancer | 4 | 2.17 | AKT2, E2F3, PIK3CB, MAPK3 | 6.76 | 0.02 | 0.15 |
| mmu04917:Prolactin signaling pathway | 4 | 2.17 | AKT2, PIK3CB, SOCS7, MAPK3 | 6.02 | 0.03 | 0.18 |
| mmu04068:FoxO signaling pathway | 5 | 2.72 | AKT2, PIK3CB, STK4, SKP2, MAPK3 | 4.10 | 0.03 | 0.20 |
| mmu05222:Small cell lung cancer | 4 | 2.17 | AKT2, E2F3, PIK3CB, SKP2 | 5.23 | 0.04 | 0.21 |
| mmu04725:Cholinergic synapse | 4 | 2.17 | AKT2, GNAI3, PIK3CB, MAPK3 | 3.89 | 0.08 | 0.30 |
| mmu05221:Acute myeloid leukemia | 3 | 1.63 | AKT2, PIK3CB, MAPK3 | 5.89 | 0.09 | 0.32 |
| mmu04722:Neurotrophin signaling pathway | 4 | 2.17 | AKT2, PIK3CB, PLCG1, MAPK3 | 3.60 | 0.10 | 0.33 |
| mmu04071:Sphingolipid signaling pathway | 4 | 2.17 | AKT2, GNAI3, PIK3CB, MAPK3 | 3.54 | 0.10 | 0.33 |

No: number of genes; %: gene percentage. Adjusted *p*: Benjamini-Hochberg *p* value.

**Table S4: Correlation between MV induced region-specific gene expression and lung volumes in LPS/MV group.**

|  | **nVt** | **nEEV** |
| --- | --- | --- |
| Antigen processing_PCA1 | 0.189 | 0.113 |
| Antigen processing_PCA2 | 0.146 | -0.188 |
| B cell activation_PCA1 | -.540^*^ | -0.274 |
| B cell receptor signaling_PCA1 | -.697^**^ | -0.149 |
| Defense response to protozoan_PCA1 | .570^*^ | 0.256 |
| Adaptive immune response_PCA1 | -.543^*^ | -0.04 |
| Adaptive immune response_PCA2 | -0.453 | -0.107 |
| Immune system process_PCA1 | 0.317 | -0.085 |
| Immune system process_PCA2 | -0.396 | -0.236 |
| Antigen processing via MHC_PCA1 | 0.282 | -0.13 |
| Immune response_PCA1 | -0.048 | -0.218 |
| Immune response_PCA2 | 0.091 | -0.212 |

Values are Pearson correlation coefficient. ^*^ *p* < 0.05, ^**^ *p* < 0.01. PCA: Principal component analysis. nVt: normalized tidal volume; nEEV: normalized EEV.

**Table S5: Comparison of MV induced region-specific gene expression in the three regions under experimental conditions.**

| **Group** | **R1** | **L2** | **R4** |
| --- | --- | --- | --- |
| Antigen processing_PCA2 | | | |
| Saline | 0.00 (0.76) | 0.00 (0.67) | 0.00 (1.30) |
| LPS | 1.08 (0.67) | 0.93 (0.72) | 0.82 (0.72) |
| MV | 1.82 (0.46)^**^ | 1.65 (0.34)^**^ | 1.28 (0.28) |
| LPS/MV | 1.44 (1.27)^*^ | 1.00 (1.00) | 1.25 (1.00) |
| Adaptive immune response_PCA2 | | | |
| Saline | 0.00 (1.18) | 0.00 (1.20) | 0.00 (1.77) |
| LPS | 1.07 (0.54) | 0.99 (0.38) | 0.85 (0.47) |
| MV | 1.93 (0.62)^**^ | 1.92 (0.39)^***^ | 1.42 (0.54) |
| LPS/MV | 1.16 (0.30) | 1.12 (0.16)^*^ | 1.32 (0.47) |
| Immune system process_PCA2 | | | |
| Saline | 0.00 (1.16) | 0.00 (1.10) | 0.00 (1.74) |
| LPS | 0.92 (0.61) | 0.82 (0.57) | 0.75 (0.61) |
| MV | 2.14 (0.53)^*** #^ | 1.93 (0.40)^***^ | 1.38 (0.49) |
| LPS/MV | 0.87 (0.38) | 0.74 (0.17) | 1.00 (0.45) |
| Antigen processing via MHC_PCA1 | | | |
| Saline | 0.00 (0.06) | 0.00 (0.10) | 0.00 (0.20) |
| LPS | 2.05 (0.06)^*** #^ | 2.07 (0.09)^*** #^ | 1.93 (0.06)^***^ |
| MV | 0.36 (0.10)^**^ | 0.43 (0.11)^***^ | 0.31 (0.11)^**^ |
| LPS/MV | 2.24 (0.24)^***^ | 2.24 (0.16)^***^ | 2.23 (0.15)^***^ |
| Immune response_PCA2 | | | |
| Saline | 0.00 (0.78) | 0.00 (0.65) | 0.00 (1.26) |
| LPS | 1.91 (0.50)^**^ | 1.68 (0.57)^***^ | 1.46 (0.41)^*^ |
| MV | 1.71 (0.38)^** ##^ | 1.59 (0.25)^** #^ | 1.13 (0.23) |
| LPS/MV | 2.15 (0.97)^***^ | 1.71 (0.73)^***^ | 1.97 (0.71)^**^ |

Values are mean (SD). PCA: PCA: Principal component analysis. ^*^ *p* < 0.05, ^**^ *p* < 0.01, ^***^ *p* < 0.001, compared to respective control region; ^#^ *p* < 0.05, ^###^ *p* < 0.001, compared to R4.
